# Performance and microbial community variations of anaerobic digesters under increasing tetracycline concentrations

**DOI:** 10.1007/s00253-017-8253-1

**Published:** 2017-04-01

**Authors:** Yanghui Xiong, Moustapha Harb, Pei-Ying Hong

**Affiliations:** King Abdullah University of Science and Technology (KAUST), Water Desalination and Reuse Center (WDRC), Biological and Environmental Science & Engineering Division (BESE), Thuwal, 23955-6900 Saudi Arabia

**Keywords:** Anaerobic digestion, Tetracycline, Methanogenic pathway, Propionic acid, Tetracycline resistance genes

## Abstract

The impact of different concentrations of tetracycline on the performance of anaerobic treatment was evaluated. Results revealed that for all of the tested tetracycline concentrations, no major sustained impact on methane production was observed. Instead, a significant increase in propionic acid was observed in the reactor subjected to the highest concentration of tetracycline (20 mg/L). Microbial community analyses suggest that an alternative methanogenic pathway, specifically that of methanol-utilizing methanogens, may be important for ensuring the stability of methane production in the presence of high tetracycline concentrations. In addition, the accumulation of propionate was due to an increase in volatile fatty acids (VFA)-producing bacteria coupled with a reduction in propionate utilizers. An increase in the abundance of tetracycline resistance genes associated with ribosomal protection proteins was observed after 30 days of exposure to high concentrations of tetracycline, while other targeted resistance genes showed no significant changes. These findings suggest that anaerobic treatment processes can robustly treat wastewater with varying concentrations of antibiotics while also deriving value-added products and minimizing the dissemination of associated antibiotic resistance genes.

**Electronic supplementary material:**

The online version of this article (doi:10.1007/s00253-017-8253-1) contains supplementary material, which is available to authorized users.

## Introduction

Anaerobic treatment offers several benefits over aerobic processes, including lower electricity consumption, reduced sludge production, and conversion of organic carbon to bio-energy (Batstone and Virdis 2014; McCarty and Rittmann 2001; Tchobanoglous et al. 2003; WERF 2011). The introduction of membranes to anaerobic digesters has served to further enhance the removal of organic carbon so as to reliably meet discharge or reuse requirements (Smith et al. 2013; Wei et al. 2014). These benefits offered by anaerobic treatment processes align well with both financial viability and environmental sustainability and hence demonstrate its potential to become a mainline treatment option for both municipal and industrial wastewaters.

However, an emerging concern surrounding wastewater treatment is the presence of antibiotics in influent wastewater streams. Globally, over 73 billion dose units of antibiotics are sold in retail and hospital pharmacies (Van Boeckel et al. 2014) and up to 63,100 tons of antibiotic were consumed by livestock in 2010 (Van Boeckel et al. 2015). Antibiotics that are consumed but not taken up by the hosts are excreted into wastewaters at varying concentrations. To exemplify, domestic wastewaters typically contain low concentrations of approximately 1 μg/L of tetracycline (Yang et al. 2005) while over 100 μg/L of tetracycline can be detected in hospital wastewater (Pena et al. 2010). These hospital wastewaters are combined with domestic sources in sewer systems for subsequent treatment in a municipal WWTP (Carraro et al. 2016). Additionally, livestock production farms rely heavily on antibiotics like tetracycline for therapeutic and growth promotion purposes. The rampant use of tetracycline is exhibited in the high concentrations of up to 23 mg tetracycline detected per kilogram of animal manure (Martínez-Carballo et al. 2007).

Tetracycline is a broad-spectrum antibacterial agent that exhibits activity against gram-positive and gram-negative bacteria, and is hence extensively used as a therapeutic agent in human and animal infections. Tetracycline is also used at sub-therapeutic levels for prophylactic and growth promotion purposes. When taken up by a bacterial cell, tetracycline inhibits protein synthesis through the prevention of aminoacyl-tRNA interaction with the ribosome. Given the vital role microbes play in anaerobic digestion, studies characterizing the anaerobic microbial communities in the presence of antibiotics are essential to determining the potential impacts on methane and volatile fatty acids (VFA) production. Various studies have reported a general reduction in biogas volumes, primarily that of methane, when anaerobic reactors were exposed to antibiotics (Álvarez et al. 2010; Bauer et al. 2014; Cetecioglu et al. 2015a; Cetecioglu et al. 2013). When volatile fatty acids (VFAs) were further added as substrates, utilization rates were reduced and an accumulation of propionate was observed (Cetecioglu et al. 2015a; Cetecioglu et al. 2013; Cetecioglu et al. 2015b). Lower abundance of functional genes related to acetogenesis and methanogenesis were quantified by qPCR (Cetecioglu et al. 2015a), suggesting perturbations in the anaerobic microbial communities when reactors were subjected to high concentrations of antibiotics.

Contradictory observations were, however, reported where inhibitory effects on methane generation were not observed for some types of antibiotics (e.g., tetracycline) or until certain threshold concentrations of antibiotics were present (Lallai et al. 2002; Spielmeyer et al. 2015). A separate study also showed that low concentrations of antibiotics (i.e., in the range of μg/L), representative of those found in municipal wastewaters, would result in minimal impact on overall COD removal efficiency and methane gas generation (Harb et al. 2016). It has been observed that discrepancies in impact can arise due to differences in the inoculum used and whether a sufficient acclimation period was given for the inoculum to achieve steady-state operation after exposure to antibiotics (Álvarez et al. 2010). Most of the studies previously mentioned were generally focused on the intermediate or end products, and provided insufficient details to determine if the microbial consortium had fully acclimated to the antibiotic exposure. Aydin et al. (2015) observed that addition of sulfamethoxazole, erythromycin, and tetracycline at 15–20, 1.5, and 1.5 mg/L concentrations, respectively, could produce 400 to 600 mg/L of volatile fatty acids (VFA). Similarly, Cetecioglu et al. (2015a) noted that increasing the concentrations of sulfamethoxazole to 45 mg/L could cause VFA to accumulate to a range of 35 to 438 mg/L in an anaerobic sequencing batch reactor (AnSBR).

VFAs, otherwise referred to as short chain fatty acids (SCFAs) that include acetate, butyrate, and propionate, are intermediate and/or end products of the digestion process, and their concentrations have been observed to correlate with overall functionality of the anaerobic treatment process (Ahring et al. 1995; Hino et al. 1993; Lin 1993; Varel et al. 1977) In recent years, VFAs have been identified as useful sources for the subsequent production of valuable bio-products (e.g., biopolymers, biofuels, and reduced chemicals of high values, such as alcohols, aldehydes, ketones, and esters) (Bhatia et al. 2015; Khan et al. 2016; Setiadi et al. 2015). Given the potential value of produced VFAs and their direct relationship with the functionality of the anaerobic digestion process, a further understanding of their accumulation in wastewater treatment systems is necessary. One previous study determined that when antibiotics were added at low concentrations to mice feed, gut microbiota with increased metabolic activity were observed along with a higher proportion of calorie extraction from complex carbohydrates, resulting in increased SCFA concentrations within the murine colon (Cho et al. 2012). These results suggest a potential link between antibiotic presence and changes to the anaerobic consortium that could result in higher VFAs production. However, corresponding variation of microbial community and VFA concentrations had not been characterized in-depth by earlier studies.

Drawing on the findings from prior studies, it is hypothesized that anaerobic digestion processes are sufficiently robust and stable to treat wastewaters with varying concentrations of antibiotics while also deriving different forms of valuable byproducts. Specifically, antibiotics would have negligible or even positive impacts on the long-term production of methane and VFAs during anaerobic wastewater treatment. To evaluate this hypothesis, three batch reactors, each dosed with varying concentrations of tetracycline representative of those found in wastewaters generated by municipal households, hospitals, and livestock, were operated alongside a control reactor. Anaerobic digestion performance was evaluated by measuring methane and carbon dioxide volumes, VFA concentrations, and microbial activity. Microbial community analysis by means of high-throughput sequencing was performed to relate the roles of keystone microbial populations to reactor performance. Quantification of tetracycline resistance genes was also performed to determine if the benefits associated with tetracycline-induced increases in biogas and VFA generation would be offset by the emergence of tetracycline resistance.

## Materials and methods

### Reactors operation and sampling

Anaerobic sludge was obtained from a well-performing anaerobic lab-scale CSTR reactor that had not been exposed to any antibiotics. Sludge inoculum of 100 mL each was added to four 1.2 L dark glass bottles. Nine hundred milliliters of synthetic wastewater (Nopens et al. 2001) containing 690 mg/L COD was added to the sludge to achieve approximately 1.5 to 1.6 g/L of mixed liquor volatile suspended solids (MLVSS) in each bottle (total digester volume of 1 L). The sampling event was done once in every 3-day interval. Assuming steady-state degradation, this would equate to a daily organic loading of 0.23 kg COD/(m^3^∙day), and is representative of a low-strength municipal wastewater. At each sampling event, a volume of fresh synthetic feed was also added to the batch reactors to replace the sampled volume and to maintain 0.23 kg COD/(m^3^∙day) organic loading. The headspace in each bottle was flushed with nitrogen gas, and sealed to achieve anaerobic conditions. All batch reactors were then incubated at 37 °C to simulate the typical temperature in Saudi Arabia, and continuously mixed at 100 rpm for sludge acclimation. Biogas volume and methane percentage were monitored regularly during the acclimation phase for all batch reactors. Upon digester acclimation, which is defined by stable methane gas generation that was not significantly different across all four reactors, different amounts of tetracycline hydrochloric acid (TC-HCl) were then added to each reactor. The first reactor served as control with no TC-HCl added. The remaining reactors were individually added with 1 μg/L, 150 μg/L, and 20 mg/L to approximate the concentrations of TC-HCl present in domestic wastewater (Yang et al. 2005), hospital wastewater (Pena et al. 2010), and livestock wastewater (Álvarez et al. 2010), respectively. The four reactors with 0 μg/L, 1 μg/L, 150 μg/L, and 20 mg/L of TC-HCl are subsequently referred to as R1, R2, R3, and R4, respectively. pH was maintained at 7.2 with 1 N of NaOH and 3 N of HCl in all reactors. Fifty milliliters of homogenized sludge was sampled from each reactor at 3-day intervals over 1 month, and was replaced with an equal volume of 20× synthetic wastewater with TC-HCl corresponding to the intended amount within each reactor. All sampling events were operated in an anaerobic chamber (AIRLOCK, COY laboratory products, US) with 1.5% H_2_ and 98.5% N_2_ headspace.

### Reactor performance

Reactor performance was evaluated by monitoring for the CH_4_ and CO_2_ volumes based on procedures described previously (Banks et al. 2011). COD sample was prepared by centrifugation of the suspension, and dissolved COD in the supernatant was measured with either HACH LCK 314 (15–150 mg/L) or LCK 514 COD (100–2000 mg/L) cuvette test vials (Hach-Lange, Manchester, UK) depending on the concentration to be measured, and then measured by a HACH DR2800 Spectrophotometer (Hach, Loveland, Colorado, USA). Presence of VFA was also measured for each of the reactors. For VFA concentration determination, 5 mL of the sampled sludge was first centrifuged at 5000*g*, and the supernatant filtered through a 0.22-μm cellulose acetate syringe filter. Filtrate was acidified with phosphoric acid by adding 0.1 mL of 10% phosphoric acid to every 0.9 mL of filtrate (Ibrahim et al. 2014). VFAs were measured by gas chromatography 890A (Agilent Technologies, Santa Clara, CA) coupled with a Flame Ionization Detector (FID) and J&W DB-WAX GC column (Agilent Technologies, Santa Clara, CA). The oven temperature was ramped up from 70 to 180 °C at an incremental rate of 9 °C per min. The heater temperature of FID was constant at 250 °C. The helium gas flow rate through the column was 1.5 mL/min, and the flow rate of H_2_ and air for FID was 40 and 400 mL/min, respectively. Each sample was injected in triplicate. Acetic acid, propionic acid, and butyric acid (Sigma-Aldrich, St Louis, MO) of known concentrations were analyzed in the same conditions as samples to obtain a corresponding standard curve.

### Determination of TC-HCl

Thirty to thirty-five milliliters of the sampled sludge from each reactor was centrifuged at 4000*g* for 20 min to separate the liquid and solid phases. To extract TC-HCl from the liquid phase, supernatant was filtered through a 0.22-μm cellulose acetate syringe filter, and the filtrate stored in an amber glass bottle. Twenty milliliters of Na_2_EDTA-McIlvaine buffer, prepared by mixing 19.2 g of citric acid, 17.75 g of Na2HPO4, and 60.5 g of Na2EDTA in 1.625 L of deionized water, was added to the amber glass bottle prior to solid-phase extraction (SPE). TC-HCl was extracted from the solid phase using a modified protocol as described previously (Huang et al. 2013). After SPE, sample from liquid and solid phases were analyzed on LC-MS/MS to determine tetracycline concentrations. Detailed protocol and procedure for both SPE and quantification of tetracycline concentrations can be found in the Supplementary Information.

### ATP analysis

One milliliter of fresh sludge from each anaerobic reactor was sampled and centrifuged at 3600*g* for 20 min (Centrifuge 5424R, Eppendorf, Germany). The pellet was resuspended in 20 mL of deionized water, and 50 μL of aliquot was measured for the ATP content by Celsis amplified-ATP reagent kit on an Advance Luminometer (Celsis, Westminster, London, UK). Deionized water was used as the negative control.

### DNA extraction and 16S rRNA gene-based next generation sequencing analysis

A total of 56 samples (*n* = 14 from each reactor) was collected for the entire experiment, and used for extraction of the microbial DNA. To prepare samples for DNA extraction, 1 mL of sample was centrifuged at 3400*g* for 5 min. The supernatant was discarded and the centrifuged biomass was used for DNA extraction. DNA was extracted and prepared for 16S rRNA gene-based next generation sequencing based on procedures described previously (Cheng et al. 2016) and listed in the Supplementary Information.

Prior to sequence analyses, sequences were processed for quality by trimming away adaptors and primer sequences, and removing sequences of lengths <300 nt. After chimeric sequences were removed via UCHIME, a sequencing depth of approximately 11,693 ± 4529 sequences was obtained per sample. The sequences that passed the quality control checks (>93% of the total sequences) were analyzed by two methods, namely RDP Classifier and OTU-based methods as specified in a previous study (Harb et al. 2015). Further elaboration of both methods was provided in the Supplementary Information. After assigning the phylogenetic identities using RDP Classifier, the extent of similarities among microbial communities was displayed on a non-metric multidimensional scaling (nMDS) plot. The nMDS plot was created by square-root transformation of the relative abundance of the bacterial and archaeal genera obtained from RDP Classifier analysis, and then computed for Bray-Curtis similarities. The measured parameters related to the performance of anaerobic digestion, i.e., volume of CH_4_ and CO_2_, concentration of propionic and acetic acid were also collated, log-transformed, and normalized for principal component analysis (PCA). Based on the VFA vs. time profile, the rate of increment for VFA in R4 was different before and after 21 days of reactor operation. The variation of microbial community was therefore divided into two phases, termed as R4_B21_ and R4_A21_, to represent samples collected before and after day 21, respectively. All statistical analysis mentioned here was performed on Primer-7 software (Clarke and Gorley 2015). All high-throughput sequencing data were deposited in the short Read Archive (SRA) of the European Nucleotide Archive (ENA) under study accession number PRJEB17854.

### Quantitative PCR to determine the tetracycline resistance genes

Quantitative PCR (qPCR) standards of tetracycline resistance genes (tetW, tetQ, tetZ, and tetG) were prepared as described previously (Al-Jassim et al. 2015). All primers used in this study were listed in Table S2. Plasmids were diluted in series to obtain standard solutions within the range of 10^3^–10^10^ copies/μL for the respective standard curves. qPCR was conducted on Applied Biosystem® 7900HT Fast Real-Time PCR system with 96-well block module (Thermo Fisher Scientific, Carlsbad, CA, USA) similar to earlier procedures (Al-Jassim et al. 2015). Amplification efficiencies ranged between 93.4 and 103.3%, with average *R* ^2^ of more than 0.98. All NTCs have *C*_q_ values that were either of undetermined values or were higher than 36.

### Statistical analysis

All samples were measured in duplicate or in triplicate in this study. To examine the differences among sample sets, an unpaired two-tailed *t* test was conducted with an assumption of unequal variance between sample sets.

## Results

### Decrease in tetracycline hydrochloric acid, MLVSS, and microbial activity in R4 upon exposure to high tetracycline concentration

**Fig. 1:**
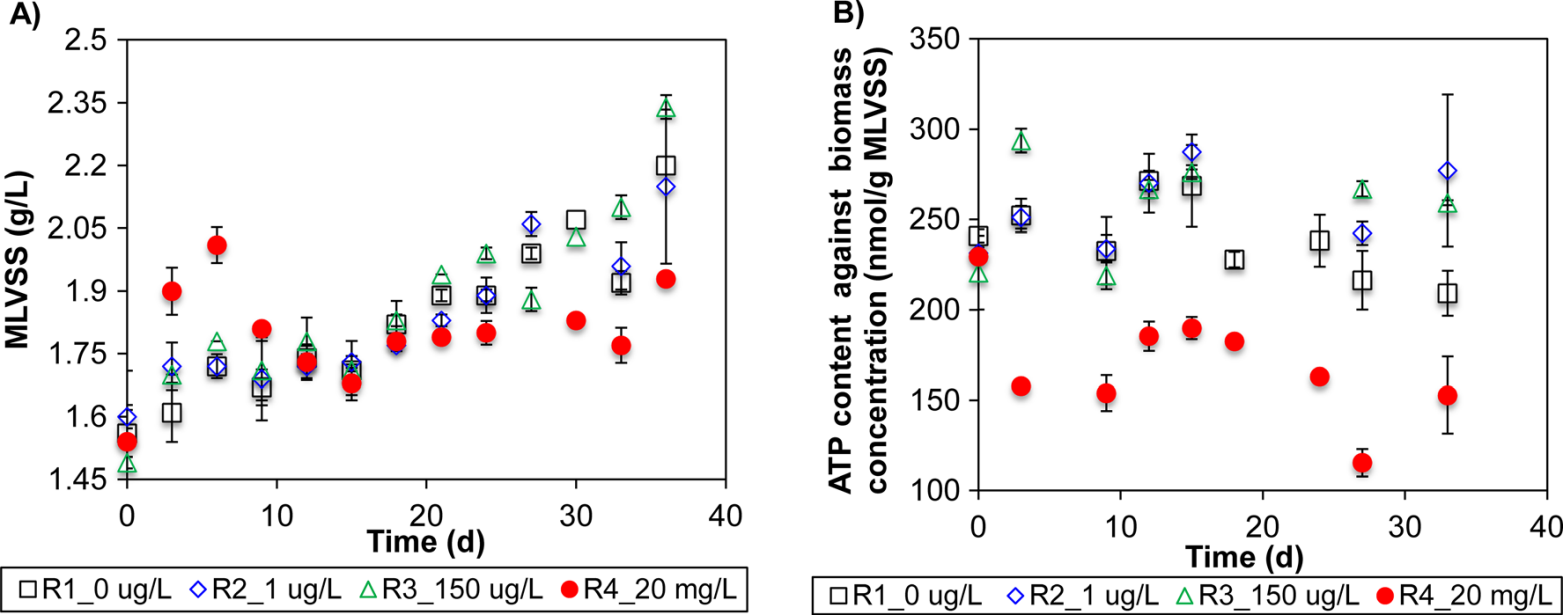
Reactor stability and activity as evaluated based on **a)**biomass concentration (MLVSS) and **b** ATP amount against biomass concentration in the digesters R1–R4 (0, 1 μg/L, 150 μg/L, and 20 mg/L of tetracycline hydrochloric acid, respectively). The *vertical bars* associated with each data point reflect the standard deviation (*n* = 2)

Total tetracycline hydrochloric acid (TC-HCl) was substantially reduced with an overall removal efficiency of 85 ± 2% in digesters R2 to R4 (Fig. S1). In R4, the total TC amount sharply decreased from 20 to 9 mg/L on day 3, and finally stabilized at 3 mg/L from day 21 onwards. Mixed liquor volatile suspended solid (MLVSS) concentrations in R1, R2, and R3 were observed to increase from 1.5 to 2.1 g/L at similar rates and were not significantly different across these three reactors (*p* > 0.05) (Fig. 1a). In R4, MLVSS increment followed a less consistent trend. To illustrate, the MLVSS in R4 sharply increased after the addition of 20 mg/L TC-HCl to above 2 g/L at day 3, and decreased back to 1.7 g/L at day 12. After day 12, MLVSS concentration in R4 gradually increased at a lower overall rate than the other three reactors to result in a final concentration of 1.9 g/L. Specific microbial activity as monitored by ATP concentrations per gram of MLVSS indicated no apparent differences among R1, R2, and R3 (*p* > 0.05) (Fig. 1b). ATP concentration was significantly reduced by 29% in R4 compared to control R1 (*p* < 0.05).

### Change in biogas composition upon exposure to high tetracycline concentration

**Fig. 2:**
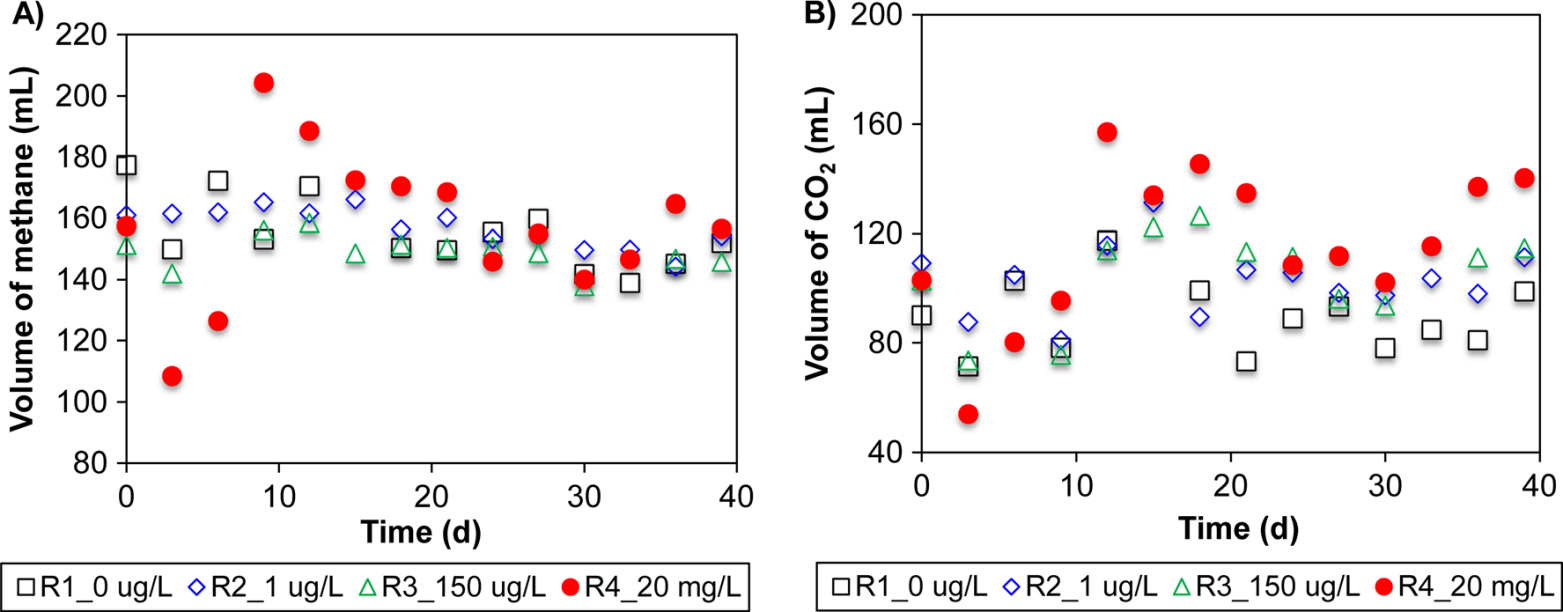
Biogas collected from the different reacto rs were analyzed for changes in methane and carbon dioxide volumes. Collected **a** methane and **b** CO_2_ per each sampling event in the digesters R1–R4 (0, 1 μg/L, 150 μg/L, and 20 mg/L of tetracycline hydrochloric acid, respectively)

The mean volume of methane produced in all R1 to R3 was 154 ± 4 mL, and values observed in R2 and R3 were not significantly different from those in control R1 (both *p* > 0.05) (Fig. 2a). For R4, the volume of methane produced immediately decreased from 160 to 110 mL at the second sampling event. Thereafter, there was an increase to 200 mL of methane on day 9 before stabilizing at 151 ± 9 mL from day 24 onwards. CO_2_ production in R2 and R3 was 17% higher than that observed in the control R1, while CO_2_ production in R4 was 29% higher than the control (Fig. 2b). The amount of CO_2_ generated in R2–R4 was significantly higher than the control (all *p* < 0.05).

### Accumulation of propionic and acetic acids upon exposure to high tetracycline concentration

**Fig. 3:**
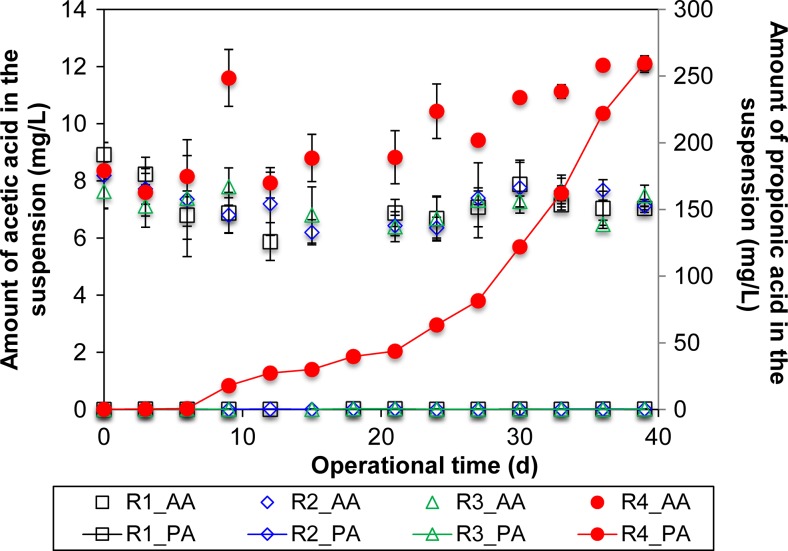
The concentrations of acetic acid (AA) and propionic acid (PA) versus time in the digesters R1–R4 (0, 1 μg/L, 150 μg/L, and 20 mg/L of tetracycline hydrochloric acid, respectively). The *vertical bars* associated with each data point reflect the standard deviation (*n* = 3)

The production of acetic and propionic acids was not affected by ≤150 μg/L TC-HCl, and the concentrations of these VFAs were not significantly different from that in control R1 (*p* > 0.05) (Fig. 3). However, both acetic and propionic acids were significantly higher in R4 (both *p* < 0.05). Specifically, propionic acid increased according to two phases. Phase 1 occurred before day 21, and it was observed that the propionic acid concentration increased at a rate of 2.9 mg/L/day, from 0.7 mg/L on day 6 to 44 mg/L on day 21. Thereafter, the rate of increment for propionic acid was 12 mg/L/day, and the measured concentration on day 39 was 260 mg/L.

### Changes of COD conversion products in biomass and VFA for highest antibiotic concentration

COD concentrations in R1, R2, and R3 were on average 208 ± 17 mg/L, and not significantly different among these three reactors (all *p* > 0.05) (Fig. S2). However, COD concentrations in R4 increased from 200 mg/L at the beginning of operation to >500 mg/L during the last 10 days of operation (*p* < 0.05).

In order to account for the total COD added to each reactor and to determine the differences between each of the four reactors in terms of COD utilized, a total COD equivalents mass balance was performed. This calculation incorporated biomass degradation and accumulation, VFA production, methane generation, and residual COD as part of an overall analysis, as shown in Table 1. The analysis was performed as a total mass balance over the entire operational period (14 sampling events) due to the cumulative nature of several of the fermentation products including MLVSS and propionic acid. Total COD input (0.69 g per sampling event) was the same across all four digesters except for R4, which had 0.21% higher COD input than the other reactors. This is due to the addition of 20 mg/L TC-HCl, which could have served as a potential substrate as shown from earlier studies that demonstrated the biodegradability of TC (Álvarez et al. 2010; Cetecioglu et al. 2014). COD converted to biomass was calculated based on sludge wasted per sampling event, average microbial degradation/regeneration rates, and biomass accumulated. As such, this value was lowest for R4 due to a lower level of MLVSS increment over the operational period (Fig. 1a). Details of the basis of calculation for this COD balance are provided in Table S1.

**Table 1:** Mass balance of total COD input and total COD output as accounted for by produced biomass, propionic acid, methane, and residual COD in digesters R1, R2, R3, and R4.

	R1	R2	R3	R4
Total COD input (g)	9.66	9.66	9.66	9.68^b^
Biomass generated (g)	0.64	0.55	0.85	0.38
COD converted to biomass (g)	3.59	3.47	3.89	3.22
COD converted to propionic acid (g)	0	0	0	0.40
COD converted to methane (g)	5.68	5.76	5.44	5.76
COD remaining in suspension^a^ (g)	0.20	0.30	0.23	0.30
Total COD output (g)	9.49	9.53	9.56	9.69

^a^COD not accounted for by propionic acid

^b^Taking into account TC-HCl as possible substrate

Based on these calculations, it was observed that the COD converted to methane was similar across the four reactors with the exception of R3, which was 5% lower overall (Table 1). However, this difference was considered relatively minor, as it was lower than the standard deviation of the average methane production from the non-tetracycline exposed reactor (*σ* = 7.4%). As described previously, the only reactor with a significant accumulation of VFAs was R4 (Fig. 3), with about 0.4 g of COD converted to propionic acid at the consequential loss of MLVSS (i.e., biomass).

### Performance parameters of R4 were different from the other reactors

**Fig. 4:**
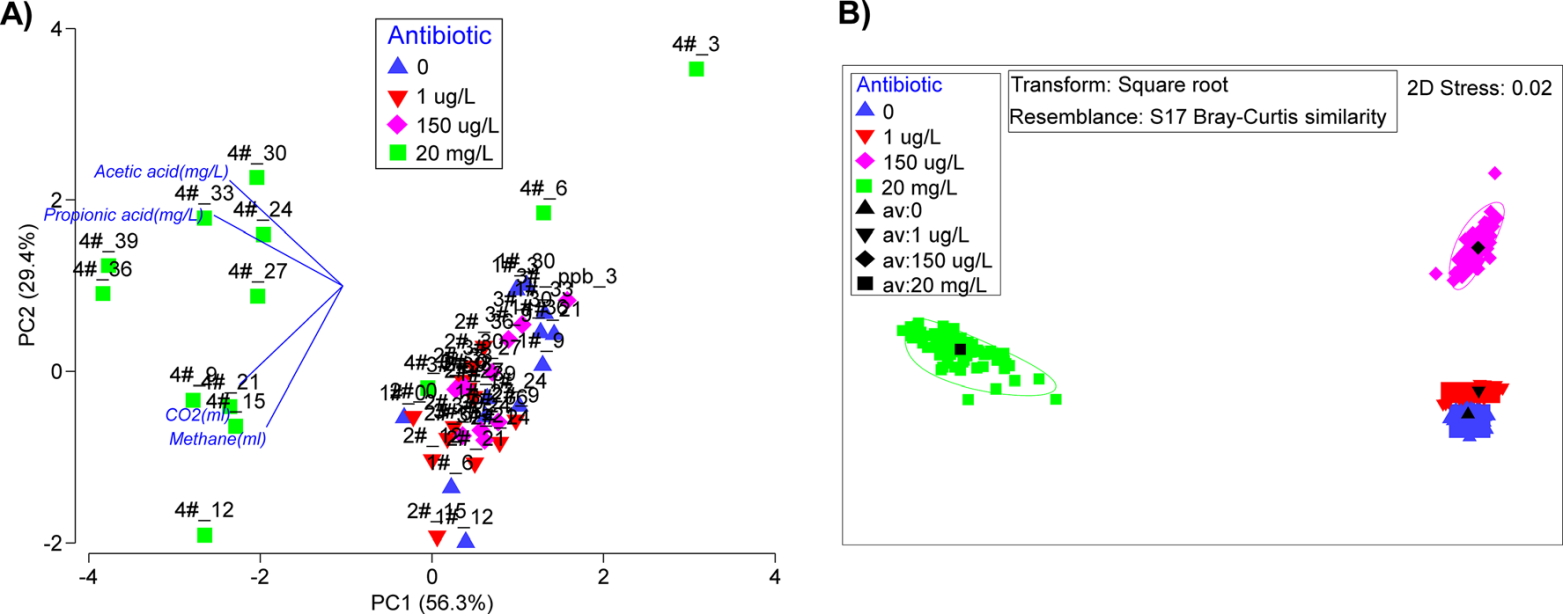
Ordination analysis of samples from digesters R1–R4 (0, 1 μg/L, 150 μg/L, and 20 mg/L of tetracycline hydrochloric acids, respectively). **a** Principal component analysis (PCA) of acetic acid, propionic acid, CO_2_, and methane. **b** Non-metric multidimensional scaling plot (nMDS, via bootstrapped averages) of microbial community

The collated data on biogas and VFA production for all four reactors was statistically analyzed on a principal component analysis (PCA) (Fig. 4a). Samples from R4 were spatially clustered apart from R1 to R3 along principal component (PC) axis 1, which accounted for 56.3% of the total variance. Samples collected from R4 were further separated into two sub-clusters along PC axis 2, with the exception of two outlier samples collected on days 3 and 6. The first sub-cluster included samples collected from days 9 to 21, while samples collected in the latter operating period formed a separate sub-cluster. Vector analysis further showed that the first sub-cluster of R4 had a comparatively lower amount of methane and CO_2_, while the second sub-cluster had higher concentrations of propionic and acetic acid. All four parameters contributed as main vectors responsible for the spatial differentiation of R4 samples from the other three reactors.

### Microbial communities at the phyla level exhibited differences among reactors

**Fig. 5:**
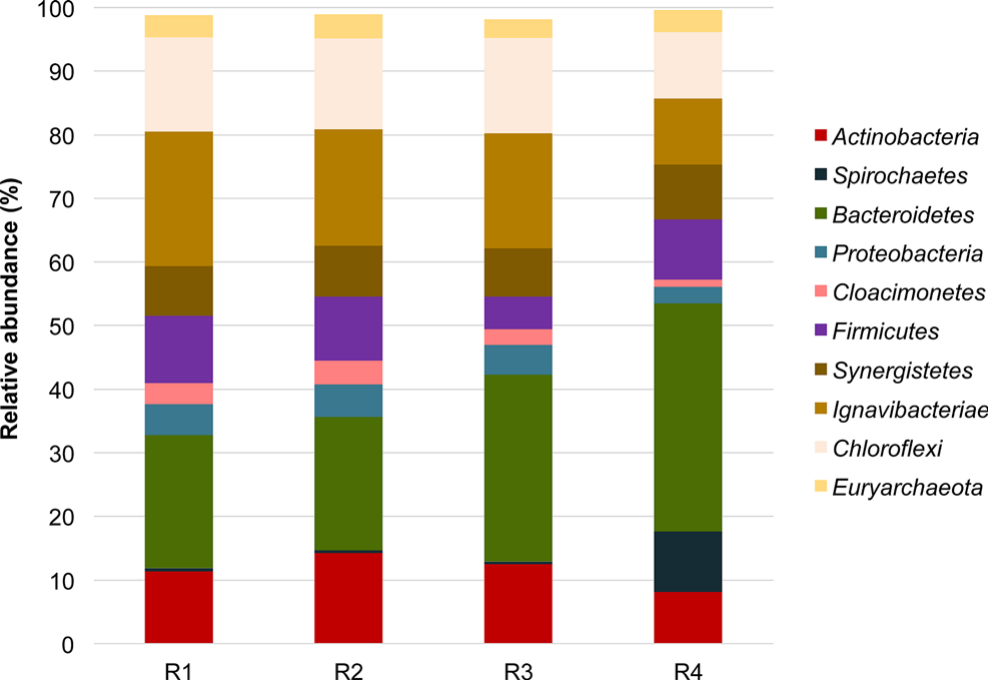
Relative abundances of bacterial phyla in digesters R1–R4 (0, 1 μg/L, 150 μg/L, and 20 mg/L of tetracycline hydrochloric acid, respectively)

The relative abundance of microbial genera across all samples were collated and statistically analyzed on a bootstrapped non-metric MDS (Fig. 4b). Exposure to 150 μg/L and the 20 mg/L concentrations of TC-HCl influenced the dynamics of bacterial populations most overtly, and resulted in a separation of the microbial communities away from those observed in R1 and R2. Several of the dominant phyla exhibited changes in relative abundance across the different reactors (Fig. 5). To illustrate, bacteria belonging to the *Bacteroidetes* phylum increased from 21.0% of the relative abundance in R1 and R2 to 29.4 and 34.2% in R3 and R4, respectively (all *p* < 0.001). *Spirochaetes* also increased significantly in relative abundance to make up 9.4% of total microbial community in R4 compared to <0.5% relative abundance in the other reactors (*p* < 0.001). Conversely, other bacterial phyla, including *Proteobacteria*, *Cloacimonetes*, *Ignavibacteriae*, and *Chloroflexi*, were shown to be significantly lower in R4 (all *p* < 0.01). Most notably, however, was the phylum *Firmicutes*, which exhibited no significant differences in the relative abundances in control R1 (10.5%) compared to R2 and R4 digesters (10.0 and 9.5%, respectively). However, R3 had a significant reduction in *Firmicutes* to 5.1% of total microbial community (*p* < 0.001).

### Changes in the relative abundance of syntrophs and methanogens among reactors

Relative abundance of genera associated with both syntrophic bacteria and methanogenic archaea were evaluated due to their integral role in the overall efficiency of anaerobic digestion (Table 2). The two most abundant syntrophic bacterial genera showed consistent relative abundances across R1 to R3, but were significantly lower in R4. To illustrate, *Syntrophorhabdus* and unclassified Syntrophobacteraceae both decreased in relative abundance from 0.25 and 0.54% in R1 to 0.13 and 0.34% in R4, respectively (*p* < 0.05). Furthermore, within R4, there was also a significant decrease in relative abundance for both *Syntrophorhabdus* and unclassified Syntrophobacteraceae in samples taken after day 21 as compared to those taken prior day 21. Conversely, higher relative abundance of *Syntrophomonas* was observed in R4 compared to the other three reactors. The relative abundance of *Syntrophomonas* significantly increased after day 21 of operation from 0.14 to 0.33% (*p* < 0.05). Among the methanogens detected, there were no significant differences in relative abundance across the reactors for a group including the acetate-utilizing *Methanothrix*, *Methanoculleus*, and *Methanobacterium* (all *p* > 0.05) with overall relative abundances of 1.2 ± 0.1%, 0.30 ± 0.03%, and 0.26 ± 0.02%, respectively. However, a higher relative abundance of *Methanomassiliicoccus* was present in R4 (0.14%) compared to the other three reactors (0.06–0.07%), and specifically, the relative abundance of *Methanomassiliicoccus* substantially increased from 0.09 to 0.19% (*p* < 0.05) after day 21 in R4. Despite not showing any significant differences across reactors, the methanogenic genus *Methanoculleus* increased in relative abundance by over one-fold after day 21 of operation in R4.

**Table 2:** Relative abundance (%) of genera identified as syntrophic or methanogenic in the anaerobic digesters (R1, R2, R3, and R4, 0, 1 μg/L, 150 μg/L, and 20 mg/L of tetracycline hydrochloride, respectively; R4_B21_ and R4_A21_ represents the samples collected from R4 before and after day 21, respectively)

	R1	R2	R3	R4	R4_B21_	R4_A21_
Unclas. Syntrophobacteraceae	0.54	0.59	0.53	0.34*	0.44*	0.20*
*Smithella*	0.03	0.03	0.02	0.02	0.02	0.02
*Syntrophorhabdus*	0.25	0.26	0.25	0.13*	0.16*	0.09*
*Syntrophomonas*	0.18	0.15	0.15	0.22*	0.14*	0.33*
*Methanobrevibacter*	0.01	0.01	0.02	0.02	0.02	0.02
*Methanobacterium*	0.27	0.28	0.23	0.25	0.24	0.26
*Methanosarcina*	0.02	0.01	0.02	0.02	0.01*	0.03*
*Methanothrix*	1.16	1.30	1.07	1.25	1.13	1.42
*Methanoculleus*	0.32	0.33	0.26	0.30	0.19*	0.44*
*Methanomassiliicoccus*	0.06	0.06	0.07	0.14*	0.09*	0.19*

An asterisk denotes that average relative abundance was significantly different (*p* < 0.05) than that of the control R1. For sample clusters R4_B21_ and R4_A21_, an asterisk indicates a significant difference in relative abundance for that particular genera when compared to each other

### Changes in relative abundance of propionate producers, propionate utilizers, and fermentative bacterium

Calculations performed based on BEST (biota and/or environment matching) analysis showed that the two variables representing propionic acid and methane production were most likely to be responsible for the spatial distribution of the microbial communities in the MDS (*ρ* = 0.337, *p* = 0.01). A number of operational taxonomic units (OTUs) known to produce VFAs were therefore further evaluated, and were observed to increase significantly in relative abundance in R4 as compared to the control. To illustrate, OTUs associated with *Clostridium aurantibutyricum*, *Microbacter margulisiae*, *Porphyromonas pogonae*, and *Treponema zuelzerae* increased in relative abundance from 0.01, 0.01, 1.72, and 0.02%, respectively, in R1 to 6.43, 2.90, 11.03, and 5.40% in R4, respectively (all *p* < 0.05) (Table 3). Additionally, two known propionate-producing bacteria showed significant increase in relative abundance in R4 after day 21. These include *Porphyromonas pogonae* and *Proteiniphilum acetatigenes*, which increased from 8.64 and 0.16% to 14.21 and 0.41%, respectively, after day 21 (*p* < 0.05). OTUs most closely related to *Syntrophobacter wolinii*, a propionate-utilizing bacterium, showed similar relative abundances in R1, R2, and R3 (i.e., 0.27, 0.28, and 0.26%, respectively), but was significantly lower in R4 at 0.14% (*p* < 0.05) and further decreased from 0.18 to 0.09% in R4 after day 21 (*p* < 0.05). OTUs associated with fermentative bacteria included *Ignavibacterium album*, *Marinithermofilum abyssi*, *Petrimonas sulfuriphila*, and *Vallitalea guaymasensis*, which decreased from 4.15, 18.11, 7.77, and 3.01%, respectively, in R1 to 1.92, 14.69, 4.31, and 0.71%, respectively, in R4 (all *p* < 0.05). These bacteria also showed significant decreases in relative abundance after day 21 in R4 (Table 3). Another fermentative species that showed significantly lower relative abundance in R4 as compared to R1 was *Macellibacteroides fermentans*, which decreased in relative abundance from 1.35% in R1 to 0.44% in R4 (*p* < 0.05). This species, however, was significantly higher in R3 than in the control, with a relative abundance of 9.27% in R3 (*p* < 0.05). Similarly, the sulfate-reducing *Desulfomicrobium baculatum* also significantly increased in relative abundance in R3 (0.24%) as compared to the control (0.08%), but was lower in R4 at 0.02% (*p* < 0.05).

**Table 3:** Relative abundance (%) of the dominant OTUs identified in anaerobic digesters with different concentrations of tetracycline hydrochloric acid (R1, R2, R3, and R4, 0, 1 µg/L, 150 µg/L, and 20 mg/L of TC-HCl, respectively; R4_B21_ represents the samples collected from digester R4 before day 21 and R4_A21_ represents those taken after day 21).

	R1	R2	R3	R4	R4_B21_	R4_A21_	Identity match (%)
*Clostridium aurantibutyricum*	0.01	0.03	0.28*	6.43*	6.19	6.74	97–99
*Microbacter margulisiae*	0.01	0.02	0.04*	2.90*	2.48	3.46	91
Porphyromonas pogonae	1.72	1.76	2.27	11.03*	8.64*	14.21*	87
*Treponema zuelzerae*	0.02	0.02	0.04	5.40*	3.71	7.65	97–99
*Proteiniphilum acetatigenes*	0.21	0.20	0.20	0.27	0.16*	0.41*	97
*Ignavibacterium album*	4.15	3.64	3.87	1.92*	2.38*	1.31*	93
*Marinithermofilum abyssi*	18.11	17.74	15.79*	14.69*	16.68*	12.04*	90
*Petrimonas sulfuriphila*	7.77	7.18	6.54*	4.31*	5.18*	3.15*	98–100
*Vallitalea guaymasensis*	3.01	2.52*	0.69*	0.71*	0.99*	0.34*	95
*Macellibacteroides fermentans*	1.35	1.87*	9.27*	0.44*	0.63*	0.20*	98
*Desulfomicrobium baculatum*	0.08	0.10	0.24*	0.02*	0.02	0.01	99
*Syntrophobacter wolinii*	0.27	0.28	0.26	0.14*	0.18*	0.09*	94
*Geobacter argillaceus*	0.86	0.81	0.68*	0.34*	0.45*	0.20*	98

An asterisk denotes that average relative abundance was significantly different (*p* < 0.05) than that of the control digester R1. For sample clusters R4_B21_ and R4_A21_, an asterisk indicates a significant difference as compared to each other

### Increased abundance of tetW and tetQ genes in R4 during the latter operational phase

**Fig. 6:**
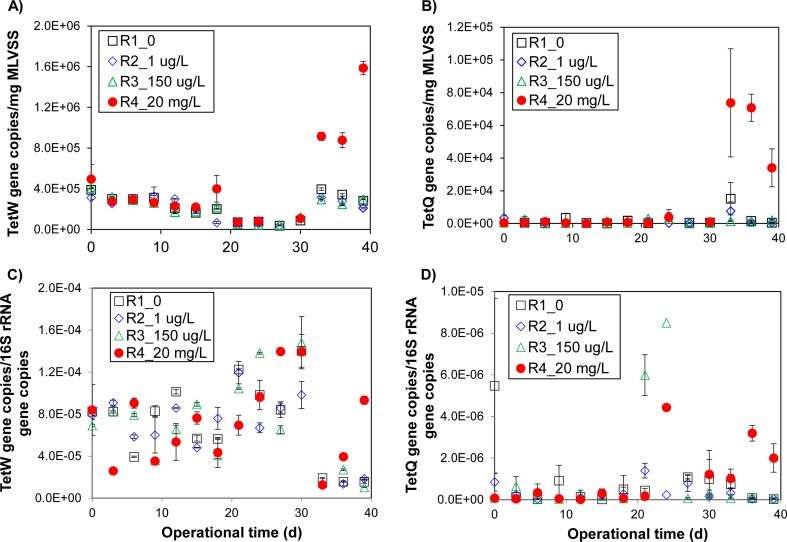
The average of abundance of tetracycline resistance genes based on different normalization methods. Abundance of **a** tetW and **b** tetQ tetracycline resistance genes normalized against mixed liquor suspended solids (MLVSS), and **c** tetW and **d** tetQ normalized against 16S rRNA copies, and their associated changes with time in the digesters R1–R4 (0, 1 µg/L, 150 µg/L, and 20 mg/L of tetracycline hydrochloric acid, respectively). The *vertical bars* associated with each data point reflect the standard deviation (*n* = 2)

Both tetW and tetQ genes confer resistance against tetracycline by the ribosomal protection protein (RPP). In R4, the abundance of tetW and tetQ genes normalized against the biomass concentration (MLVSS), increased by 3.4-fold and 115.9-fold, respectively, after day 30 (Fig. 6a, b). To illustrate, the abundance of tetW increased from an average 2.59 × 10^5^ ± 1.35 × 10^5^ copies/g MLVSS before day 30 to 1.13 × 10^6^ ± 3.99 × 10^5^ copies/g MLVSS. Similarly, the abundance of tetQ increased from an average 5.09 × 10^2^ ± 3.49 × 10^2^ copies/g MLVSS before day 30 to 5.95 × 10^4^ ± 2.21 × 10^4^ copies/g MLVSS. The abundance of both tetW and tetQ genes in R4 after day 30 were significantly higher compared to all remaining reactors (*p* < 0.10). In comparison, the average relative abundance of tetW and tetQ genes in R1 to R3 were 2.15 × 10^5^ ± 1.14 × 10^5^ copies/g MLVSS and 1.44 × 10^3^ ± 2.67 × 10^3^ copies/g MLVSS, respectively, and were not significantly different among these three reactors (*p* > 0.05). When normalized against 16S rRNA copies, higher abundances of tetW and tetQ were also present in R4 in comparison to the other reactors after day 33 (Fig. 6c, d). To illustrate, the abundances of tetW were slightly varied with an average 7.94 × 10^−5^ ± 2.35 × 10^−5^ copies/16S rRNA copies among the reactors before day 33. The average abundances of tetW were then decreased to 1.65 × 10^−5^ ± 4.81 × 10^−6^ copies/16S rRNA copies in R1–R3 and to 4.85 × 10^−5^ ± 4.11 × 10^−5^ copies/16S rRNA copies in R4. tetQ was present in R1–R4 before day 33, with an average 8.80 × 10^−7^ ± 1.81 × 10^−6^ copies/16S rRNA copies. Subsequently, the average abundance of tetQ gene increased to 2.07 × 10^−6^ ± 1.09 × 10^−6^ copies/16S rRNA copies in R4, while it decreased to 1.67 × 10^−7^ ± 2.32 × 10^−7^ copies/16S rRNA copies in R1–R3.

In contrast, tetZ and tetG, both of which confer resistance against tetracycline by the efflux pump mechanism, exhibited the same overall trend across all four reactors. The average abundance of tetZ and tetG were not significantly different regardless of whether the abundance was normalized against MLVSS or16S rRNA copies (*p* > 0.09 for all). Throughout the course of operation, the abundance of tetZ and tetG was on average 1.35 × 10^5^ ± 1.12 × 10^5^ copies/g MLVSS and 4.45 × 10^4^ ± 2.86 × 10^4^ copies/g MLVSS, respectively, in all reactors. Normalization against 16S rRNA copies showed that the average abundance of tetZ and tetG was 3.40 × 10^−5^ ± 1.67 × 10^−5^ copies/16S rRNA copies and 1.75 × 10^−5^ ± 1.74 × 10^−5^ copies/16S rRNA copies, respectively, in all reactors.

## Discussion

The present study explored the performance of anaerobic digesters under different tetracycline exposure levels. COD in suspension, ATP levels, and biomass concentrations were all not affected in the presence of tetracycline concentrations of ≤ 150 μg/L. The addition of 20 mg/L of TC-HCl, however, caused decreases in ATP and increases in COD in R4. Furthermore, unlike the increases in the biomass concentration observed in R1–R3, the biomass concentration in R4 increased at a lower rate, ultimately resulting in a lower overall biomass yield. Although in R4 biogas production initially decreased by over 30% during the first 6 days of operation before again increasing and then recovering to the expected yield levels after day 24, absolute methane production rates were not affected. These results reiterate those of previous studies in which inhibitory effects on methane generation were not observed for some types of antibiotics (e.g., tetracycline) or until certain threshold concentrations of antibiotics were present (Harb et al. 2016; Lallai et al. 2002; Spielmeyer et al. 2015).

Various other studies, however, have reported a general reduction in biogas volumes, primarily that of methane, when anaerobic reactors were exposed to antibiotics (Álvarez et al. 2010; Bauer et al. 2014; Cetecioglu et al. 2015a; Cetecioglu et al. 2013). These contradictory observations can arise due to differences in the inoculum used and/or whether a sufficient acclimation period was given for the inoculum to achieve steady-state operation after exposure to antibiotics (Álvarez et al. 2010). Acclimation allows for adaptation and selective enrichment of microbial groups that play key roles in the anaerobic fermentation process. For example, the recovery of methane yield in R4 after the initial decreases corresponded to an increase in the relative abundance of methanol-utilizing *Methanomassiliicoccus* (Dridi et al. 2012; Gorlas et al. 2012). Although there was no significant difference in the relative abundance of *Methanothrix*, *Methanoculleus*, and *Methanobacterium* among all four reactors, the relative abundance of *Methanomassiliicoccus* after day 24 remained significantly higher than that detected in the other three bioreactors. This suggests that in the presence of low concentrations of tetracycline, acetoclastic and hydrogenotrophic methanogens are likely to contribute more towards stable production of methane. Conversely, alternative methanogenic pathways may be required to achieve stable methane production in the presence of high concentrations of tetracycline. In typical anaerobic digestion, CO_2_ is generated during fermentation and then consumed by acetogenesis or hydrogenotrophic methanogenesis (Salminen et al. 2000). The increase in the methanol-utilizing methanogens, which utilize hydrogen as an electron source, from 5.1 to 9.4% of total archaeal sequences (Table S3) in R4, is possibly responsible for a shift away from CO_2_-utilizing methanogenesis and the subsequent accumulation of CO_2_ in that reactor (Fig. 2b). Nonetheless, any actual shift in methanogenic pathways cannot be determined by relative abundance alone. Furthermore, this study utilized a DNA-based sequencing approach to determine the relative abundance of methanogens and bacterial populations. DNA-based sequencing does not differentiate between dead and alive cells. Neither does this approach suffice in identifying active microbial populations. This limitation is further compounded when high antibiotic concentrations would create a hostile condition that impedes cell activities. Earlier studies have already shown that presence of bacterial populations identified by the DNA-based method may not correlate to microbial activities (De Vrieze et al. 2016; Miller et al. 2016). Therefore, the possible shift to methanol-utilizing methanogenesis in the presence of increasing antibiotic concentrations would need to be confirmed by functional gene expression through metatranscriptomics and metaproteomics.

It has been estimated that although anaerobic fermentation can produce methane, and the subsequent utilization of methane captured in headspace biogas can yield cost recoveries of 0.02–0.03 euro/m^3^, those cost recoveries are significantly lower (0.002–0.005 euro/m^3^) when total methane (i.e., biogas and dissolved methane recovery) is attempted (Pretel et al. 2015). The lower profitability of capturing total methane would mean that a significant portion of methane is released into the environment in the dissolved form through the treated effluent. This in turn poses a risk to climate change as methane is a gas with over 20 times the global warming potential of CO_2_. Given the potential drawbacks associated with methane recovery from anaerobic digestion, developing the anaerobic fermentation process to yield higher-value end products has become a topic of interest in recent years (Kleerebezem et al. 2015). Volatile fatty acids (VFAs), for example, can be used as precursors to the production of polyhydroxyalkanoates (PHAs), which have an estimated value that is five times higher than that of methane. Reported concentrations of total recoverable VFA during the anaerobic treatment of municipal wastewater range from 15 to 24 mg/L (Shin et al. 2014). These values are considered too low for any economically feasible recovery of such products from municipal wastewaters. As such, past studies have attempted to maximize the recovery of VFAs by increasing organic loading rates, which favors higher VFA extraction (Lim et al. 2008; Rincón et al. 2008). A common method to increase organic loading rates is to concentrate wastewater prior to the anaerobic digestion phase. This approach, however, also concentrates the antibiotics in the wastewater and their potential toxicity. In general, accumulation of VFA occurs during a stress situation (e.g., over-loading, presence of high concentration of heavy metal, and antibiotics) (Ahring et al. 1995; Hino et al. 1993; Lin 1993; Varel et al. 1977). As observed in the present study, an accumulation of VFAs (in this case propionic acid) was also observed at the highest tetracycline concentrations (R4). This accumulation did not, however, lead to a reduction in methane production rates, suggesting that it is potentially feasible to utilize sub-toxic concentrations of antibiotics to increase VFA yield without disrupting the anaerobic process. The levels of propionic acid produced by R4 in the present study would be equivalent to approximately 200 kg per day in a medium size full-scale anaerobic treatment system of an assumed volume of 10,000 m^3^. Given that the currently estimated cost of production of propionic acid by biological means is approximately $3.5 per kilogram (Rodriguez et al. 2014), the economic feasibility of recovering this VFA from anaerobic sludge would largely depend on the future improvement of available separation methods (Singhania et al. 2013).

The corresponding microbial characterization showed that VFA producers were significantly higher in R4 than the control reactor. Two of the OTUs associated with these VFA-producing bacteria were *Clostridium aurantibutyricum* and *Microbacter margulisiae*, which increased in relative abundance by 643-fold and 290-fold, respectively, in R4 compared to the control R1 (Table 3). Both of these bacteria species are gram-positive with the former being a spore-forming member of the phylum *Firmicutes* and the latter belonging to the order *Actinomycetales*. The ability to enter into dormancy through spore formation may have allowed *Clostridium aurantibutyricum* to tolerate the initial exposure to the 20 mg/L of tetracycline (Fig. S1) and then recover its activity to produce VFA when tetracycline concentrations reduced and stabilized at 3 mg/L of TC-HCl. Furthermore, tetracycline is a known product of actinomycete secondary metabolism (Thaker et al. 2010), and bacterial OTUs associated with this order may be inherently resistant to this antibiotic. The observed increase in VFA producers was accompanied by a decrease in propionate-utilizing bacterial OTUs such as *Desulfomicrobium baculatum* and *Syntrophobacter wolinii* (Table 3), which may explain the reduced VFA utilization rates and accumulation of propionate observed in earlier studies (Cetecioglu et al. 2015a; Cetecioglu et al. 2013; Cetecioglu et al. 2015b). However, it is important to point out that high-throughput platforms achieve only short reads and are unable to assign phylogenetic identities at species level in high confidence. Hence, future studies that involve cultivation of these bacterial species in pure microcosms to verify their roles in VFA utilization rates and propionate accumulation should be performed.

Despite the potentially positive economic impact of antibiotics on the anaerobic digestion process, changes in the corresponding abundance of antibiotic resistance genes should also be taken into consideration. It was determined that regardless of the tetracycline exposure levels, average abundances of both tetZ and tetG genes exhibited a common baseline abundance across all four reactors. Both of these genes confer resistance against tetracycline by the efflux pump mechanism. This is in contrast to the observed increase in tetW and tetQ genes after 30 days of exposure to high concentrations of tetracycline. tetW and tetQ confer resistance against tetracycline by the ribosomal protection protein (RPP), which prevents interaction between tetracycline and the ribosomes of bacterial cells (Connell et al. 2003). Higher abundances of tetracycline resistance genes related to RPP in anoxic or anaerobic ecosystems at high concentration of antibiotics have also been reported in other studies. For example, both the abundance of tetW and tetQ in anaerobic co-digestion of pig manure and wheat straw increased from 1.68 × 10^9^ to 4.92 × 10^9^ copies/g, and from 9.33 × 10^6^ to 3.43 × 10^7^ copies/g, respectively, as the oxytetracycline concentrations increased from 0 to 140 mg/kg (dry sludge) (Wang et al. 2016). Similarly, the abundance of tetQ increased by at least 1000 times, from under detectable limits to 1 × 10^3^ copies/mL of wastewater, when a cocktail of pharmaceuticals containing an initial concentration of 10 mg/L sulfamethoxazole, 0.5 mg/L erythromycin, and 0.5 mg/L tetracycline increased to 20 mg/L sulfamethoxazole, 1.5 mg/L erythromycin, and 1.5 mg/L tetracycline (Aydin et al. 2015).

In some instances, the increase in tetracycline resistance genes associated with RPP but not with efflux pumps may be due to a corresponding shift in the microbial community. tetW is commonly found in several genera, including *Clostridium*, while tetQ is commonly associated with the genera *Porphyromonas* (Liu and Pop 2009). Both of the aforementioned genes increased significantly in relative abundance in R4 after 30 days of operation (Fig. 6). Although mobile genetic elements were not quantified in this study and that the true extent of horizontal gene transfer rates remains unknown, there exists a potential for these antibiotic resistance genes to be disseminated in wastewater effluents. The potential dissemination of these genes needs to be taken into account when considering the potential advantages of antibiotics on resource recovery during long-term anaerobic treatment. Furthermore, increases in tetracycline resistance genes in the liquid fraction may also result in a corresponding increase in their abundances within the sludge biomass. However, the low sludge production rates of anaerobic processes (i.e., high SRTs) would minimize the risks associated with the disposal or further treatment of these wastes. This aspect of the anaerobic digestion process, in combination with its energy and VFA recovery potential, highlights its possible advantages for the treatment of antibiotic-containing wastewaters.

## Electronic supplementary material

ESM 1 (DOCX 102 kb)

## References

[CR1] Ahring BK, Sandberg M, Angelidaki I (1995). Volatile fatty acids as indicators of process imbalance in anaerobic digestors. Appl Microbiol Biotechnol.

[CR2] Al-Jassim N, Ansari MI, Harb M, Hong P-Y (2015). Removal of bacterial contaminants and antibiotic resistance genes by conventional wastewater treatment processes in Saudi Arabia: is the treated wastewater safe to reuse for agricultural irrigation?. Water Res.

[CR3] Álvarez J, Otero L, Lema J, Omil F (2010). The effect and fate of antibiotics during the anaerobic digestion of pig manure. Bioresour Technol.

[CR4] Aydin S, Ince B, Ince O (2015). Development of antibiotic resistance genes in microbial communities during long-term operation of anaerobic reactors in the treatment of pharmaceutical wastewater. Water Res.

[CR5] Banks CJ, Chesshire M, Heaven S, Arnold R (2011). Anaerobic digestion of source-segregated domestic food waste: performance assessment by mass and energy balance. Bioresour Technol.

[CR6] Batstone DJ, Virdis B (2014). The role of anaerobic digestion in the emerging energy economy. Curr Opin Biotechnol.

[CR7] Bauer A, Lizasoain J, Nettmann E, Bergmann I, Mundt K, Klocke M, Rincón M, Amon T, Piringer G (2014). Effects of the antibiotics chlortetracycline and enrofloxacin on the anaerobic digestion in continuous experiments. BioEnergy Res.

[CR8] Bhatia S, Yi DH, Kim HJ, Jeon JM, Kim YH, Sathiyanarayanan G, Seo H, Lee J, Kim JH, Park K (2015). Overexpression of succinyl-CoA synthase for poly (3-hydroxybutyrate-co-3-hydroxyvalerate) production in engineered *Escherichia coli* BL21 (DE3). J Appl Microbiol.

[CR9] Carraro E, Bonetta S, Bertino C, Lorenzi E, Bonetta S, Gilli G (2016). Hospital effluents management: chemical, physical, microbiological risks and legislation in different countries. J Environ Manag.

[CR10] Cetecioglu Z, Ince B, Azman S, Ince O (2014). Biodegradation of tetracycline under various conditions and effects on microbial community. Appl Biochem Biotechnol.

[CR11] Cetecioglu Z, Ince B, Gros M, Rodriguez-Mozaz S, Barceló D, Ince O, Orhon D (2015). Biodegradation and reversible inhibitory impact of sulfamethoxazole on the utilization of volatile fatty acids during anaerobic treatment of pharmaceutical industry wastewater. Sci Total Environ.

[CR12] Cetecioglu Z, Ince B, Gros M, Rodriguez-Mozaz S, Barceló D, Orhon D, Ince O (2013). Chronic impact of tetracycline on the biodegradation of an organic substrate mixture under anaerobic conditions. Water Res.

[CR13] Cetecioglu Z, Ince B, Ince O, Orhon D (2015). Acute effect of erythromycin on metabolic transformations of volatile fatty acid mixture under anaerobic conditions. Chemosphere.

[CR14] Cheng H, Xie Y, Villalobos LF, Song L, Peinemann K-V, Nunes S, Hong P-Y (2016) Antibiofilm effect enhanced by modification of 1, 2, 3-triazole and palladium nanoparticles on polysulfone membranes. Sci Reports 6 10.1038/srep24289 PMC4828667 27068576

[CR15] Cho I, Yamanishi S, Cox L, Methé BA, Zavadil J, Li K, Gao Z, Mahana D, Raju K, Teitler I (2012). Antibiotics in early life alter the murine colonic microbiome and adiposity. Nature.

[CR16] Clarke K, Gorley R (2015) PRIMER Version 7: User Manual/Turotial, vol. 192, PRIMER-E

[CR17] Connell SR, Tracz DM, Nierhaus KH, Taylor DE (2003). Ribosomal protection proteins and their mechanism of tetracycline resistance. Antimicrob Agents Chemotherapy.

[CR18] De Vrieze J, Regueiro L, Props R, Vilchez-Vargas R, Jáuregui R, Pieper DH, Lema JM, Carballa M (2016). Presence does not imply activity: DNA and RNA patterns differ in response to salt perturbation in anaerobic digestion. Biotechnol Biofuels.

[CR19] Dridi B, Fardeau M-L, Ollivier B, Raoult D, Drancourt M (2012) *Methanomassiliicoccus luminyensis* gen. nov., sp. nov., a methanogenic archaeon isolated from human faeces. Int J Sys Evolution Microbiol 62(8):1902–1907 10.1099/ijs.0.033712-0 22859731

[CR20] Gorlas A, Robert C, Gimenez G, Drancourt M, Raoult D (2012) Complete genome sequence of *Methanomassiliicoccus luminyensi*s, the largest genome of a human-associated Archaea species. J Bacteriol 194(17):4745–4745 10.1128/JB.00956-12 PMC3415480 22887657

[CR21] Harb M, Wei C-H, Wang N, Amy G, Hong P-Y (2016). Organic micropollutants in aerobic and anaerobic membrane bioreactors: changes in microbial communities and gene expression. Bioresour Technol.

[CR22] Harb M, Xiong Y, Guest J, Amy G, Hong P-Y (2015). Differences in microbial communities and performance between suspended and attached growth anaerobic membrane bioreactors treating synthetic municipal wastewater. Environmental Science: Water Res Technol.

[CR23] Hino T, Takeshi K, Kanda M, Kumazawa S (1993). Effects of aibellin, a novel peptide antibiotic, on rumen fermentation in vitro. J Dairy Sci.

[CR24] Huang Y, Cheng M, Li W, Wu L, Chen Y, Luo Y, Christie P, Zhang H (2013). Simultaneous extraction of four classes of antibiotics in soil, manure and sewage sludge and analysis by liquid chromatography-tandem mass spectrometry with the isotope-labelled internal standard method. Anal Methods.

[CR25] Ibrahim V, Hey T, Jönsson K (2014) Determining short chain fatty acids in sewage sludge hydrolysate: A comparison of three analytical methods and investigation of sample storage effects. J Environ Sci 26(4):926--933 10.1016/S1001-0742(13)60516-1 25079424

[CR26] Khan M, Ngo H, Guo W, Liu Y, Zhou J, Zhang J, Liang S, Ni B, Zhang X, Wang J (2016). Comparing the value of bioproducts from different stages of anaerobic membrane bioreactors. Bioresour Technol.

[CR27] Kleerebezem R, Joosse B, Rozendal R, Van Loosdrecht MC (2015). Anaerobic digestion without biogas?. Reviews Environ Sci Bio/Technol.

[CR28] Lallai A, Mura G, Onnis N (2002). The effects of certain antibiotics on biogas production in the anaerobic digestion of pig waste slurry. Bioresour Technol.

[CR29] Lim S-J, Kim BJ, Jeong C-M, Ahn YH, Chang HN (2008). Anaerobic organic acid production of food waste in once-a-day feeding and drawing-off bioreactor. Bioresour Technol.

[CR30] Lin C-Y (1993). Effect of heavy metals on acidogenesis in anaerobic digestion. Water Res.

[CR31] Liu B, Pop M (2009). ARDB—antibiotic resistance genes database. Nucleic Acids Res.

[CR32] Martínez-Carballo E, González-Barreiro C, Scharf S, Gans O (2007). Environmental monitoring study of selected veterinary antibiotics in animal manure and soils in Austria. Environ Pollution.

[CR33] McCarty PL, Rittmann BE (2001). Environmental biotechnology: principles and applications.

[CR34] Miller JH, Novak JT, Knocke WR, Pruden A (2016) Survival of antibiotic resistant bacteria and horizontal gene transfer control antibiotic resistance gene content in anaerobic digesters. Frontiers Microbiol 7 10.3389/fmicb.2016.00263 PMC4781833 27014196

[CR35] Nopens I, Capalozza C, Vanrolleghem P (2001). Stability analysis of a synthetic municipal wastewater.

[CR36] Pena A, Paulo M, Silva L, Seifrtová M, Lino C, Solich P (2010). Tetracycline antibiotics in hospital and municipal wastewaters: a pilot study in Portugal. Anal Bioanal Chem.

[CR37] Pretel R, Shoener B, Ferrer J, Guest J (2015). Navigating environmental, economic, and technological trade-offs in the design and operation of submerged anaerobic membrane bioreactors (AnMBRs). Water Res.

[CR38] Rincón B, Sánchez E, Raposo F, Borja R, Travieso L, Martín M, Martín A (2008). Effect of the organic loading rate on the performance of anaerobic acidogenic fermentation of two-phase olive mill solid residue. Waste Manag.

[CR39] Rodriguez BA, Stowers CC, Pham V, Cox BM (2014). The production of propionic acid, propanol and propylene via sugar fermentation: an industrial perspective on the progress, technical challenges and future outlook. Green Chem.

[CR40] Salminen E, Rintala J, Lokshina LY, Vavilin V (2000). Anaerobic batch degradation of solid poultry slaughterhouse waste. Water Sci Techn.

[CR41] Setiadi T, Aznury M, Trianto A, Pancoro A (2015). Production of polyhydroxyalkanoate (PHA) by Ralstonia eutropha JMP 134 with volatile fatty acids from palm oil mill effluent as precursors. Water Sci Technol.

[CR42] Shin C, McCarty PL, Kim J, Bae J (2014). Pilot-scale temperate-climate treatment of domestic wastewater with a staged anaerobic fluidized membrane bioreactor (SAF-MBR). Bioresour Technol.

[CR43] Singhania RR, Patel AK, Christophe G, Fontanille P, Larroche C (2013). Biological upgrading of volatile fatty acids, key intermediates for the valorization of biowaste through dark anaerobic fermentation. Bioresour Technol.

[CR44] Smith AL, Skerlos SJ, Raskin L (2013). Psychrophilic anaerobic membrane bioreactor treatment of domestic wastewater. Water Res.

[CR45] Spielmeyer A, Breier B, Groißmeier K, Hamscher G (2015). Elimination patterns of worldwide used sulfonamides and tetracyclines during anaerobic fermentation. Bioresour Technol.

[CR46] Tchobanoglous G, Burton FL, Stensel HD, Metcalf, Eddy I (2003). Wastewater engineering: treatment and reuse Inc.

[CR47] Thaker M, Spanogiannopoulos P, Wright GD (2010). The tetracycline resistome. Cell Mol Life Sci.

[CR48] Van Boeckel TP, Brower C, Gilbert M, Grenfell BT, Levin SA, Robinson TP, Teillant A, Laxminarayan R (2015). Global trends in antimicrobial use in food animals. Pro Nation Acad Sci.

[CR49] Van Boeckel TP, Gandra S, Ashok A, Caudron Q, Grenfell BT, Levin SA, Laxminarayan R (2014). Global antibiotic consumption 2000 to 2010: an analysis of national pharmaceutical sales data. Lancet Infect Diseases.

[CR50] Varel V, Isaacson H, Bryant M (1977). Thermophilic methane production from cattle waste. Appl Environ Microbiol.

[CR51] Wang X, Pan H, Gu J, Qian X, Gao H, Qin Q (2016) Effects of oxytetracycline on archaeal community, and tetracycline resistance genes in anaerobic co-digestion of pig manure and wheat straw. Environ Technol:1–9 10.1080/09593330.2016.1181109 27115735

[CR52] Wei C-H, Harb M, Amy G, Hong P-Y, Leiknes T (2014). Sustainable organic loading rate and energy recovery potential of mesophilic anaerobic membrane bioreactor for municipal wastewater treatment. Bioresour Technol.

[CR53] WERF (2011) Energy production and efficiency research-the roadmap to net-zero energy.

[CR54] Yang S, Cha J, Carlson K (2005). Simultaneous extraction and analysis of 11 tetracycline and sulfonamide antibiotics in influent and effluent domestic wastewater by solid-phase extraction and liquid chromatography-electrospray ionization tandem mass spectrometry. J Chrom A.

