# Supplementary material for: Performance and microbial community variations of anaerobic digesters under increasing tetracycline concentrations
Source: Appl Microbiol Biotechnol. 2017 Apr 1;101(13):5505–17. doi: 10.1007/s00253-017-8253-1 (PMC5486833; doi:10.1007/s00253-017-8253-1)
Supplement: Supplementary file 1 — (DOCX 102 kb) [file 253_2017_8253_MOESM1_ESM.docx]

Electronic Supplementary Material for:

Applied Microbiology and Biotechnology

**Performance and microbial community variations of anaerobic digesters under increasing tetracycline concentrations**

Yanghui Xiong ^1^, Moustapha Harb ^1^, Pei-Ying Hong * ^1^

1. King Abdullah University of Science and Technology (KAUST), Water Desalination and Reuse Center (WDRC), Biological and Environmental Science & Engineering

Division (BESE), Thuwal, 23955-6900, Saudi Arabia

* Corresponding author:

Pei-Ying Hong

Email: [peiying.hong@kaust.edu.sa](mailto:peiying.hong@kaust.edu.sa)

Phone: +966-12-8082218

**Table S1.** Details of the basis of calculation for COD balance in four reactors.

**Biogas Composition Calculations**

Assuming:

Peptone = 100% Protein

Starch = 100% Carbohydrate

Milk Powder = 41.7% Carbohydrate, 29.7% Lipids, 28.6% Protein

Yeast Extract = 46.1% Carbohydrate, 9.0% Lipids, 44.9% Protein

Considering typical chemical formulas (Rittmann and McCarty, 2012)

Carbohydrates: C_6_H_10_O_5_ f_s_ = 0.28 f_e_=0.72

Proteins: C_16_H_24_O_5_ f_s_ = 0.08 f_e_=0.92

Lipids: C_16_H_32_O_2_ f_s_ = 0.06 f_e_=0.94

Acetate: C_2_H_4_O_2_ f_s_ = 0.11 f_e_=0.89

and using:

C_w_H_x_O_y_N_z_ + *a*O_2_ 🡪 *b*CO_2_ + *c*H_2_O

The overall synthetic wastewater composition (690 mg/L as COD) is calculated:

50% Carbohydrate, 19% Protein, 10% Lipids, and 21% Acetate

Based on the generalized stoichiometric equation and assuming f_s_ + f_e_ = 1 (Rittmann and McCarty, 2012, eqn. 13.5):

C_w_H_x_O_y_N_z_ + *a*H_2_O 🡪 *b*CH_4_ + *c*CO_2_ + *d*C_5_H_7_O_2_N + *e*NH_4_ + *g*HCO_3_^-^

For each M compound:

M CH_4_ M CO_2_

Carbohydrates: 2.16 2.50

Proteins: 7.60 3.35

Lipids: 9.20 1.52

Acetate: 0.89 0.93

Therefore 0.815 M of CO2 will be produced for each 1 M of CH_4_ for this synthetic wastewater.

Average biogas production from reactors (measured):

R1 (mL) R2 (mL) R3 (mL) R4 (mL)

Captured CH_4_ 2180 2200 2080 2210

Captured CO_2_ 1270 1440 1430 1619

Based on the ideal gas law, total produced CH_4_ and CO_2_ could be determined:

R1 R2 R3 R4

CH_4_ 89 (mM) 90 (mM) 85 (mM) 90 (mM)

CO_2_  52 (mM) 59 (mM) 59 (mM) 66 (mM)

CO_2_/CH_4_ 0.58 0.66 0.69 0.74

CH_4_ 1.42 g 1.44 g 1.36 g 1.44 g

**Theoretical methane production as COD**

Maximum theoretical methane production was calculated based on:

CH_4_ + *2*O_2_ 🡪 CO_2_ + *2*H_2_O

(16g) + (64g) 🡪 (44g) + (36g)

0.25 g CH_4_ = 1 g COD

Therefore ideally at STP, 1 g COD = 350 mL CH_4_.

R1 R2 R3 R4

CH_4_ 1.42 g 1.44 g 1.36 g 1.44 g

COD _CH4_ 5.68 g 5.76 g 5.44 g 5.76 g

**COD as Propionate**

CH_3_(CH_2_)COOH + *3.5*O_2_ 🡪 *3*CO_2_ + *3*H_2_O

(74g) + (112g) 🡪 (132g) + (54g)

Therefore: 1 g propionate = 1.51 g COD

COD as propionate (R4) = COD _propionate_ = 0.27 g propionate x 1.51 = 0.40 g COD

R1 R2 R3 R4

COD _propionate_ 0 g 0 g 0 g 0.40 g

**COD as Tetracycline**

C_22_H_24_N_2_O_8_ MW = 444.4 g/mol MW (C) = 264 g/mol

TOC = 0.595 g/g C_22_H_24_N_2_O_8_

COD / TOC = 3 (for water and wastewater) sorption coefficient = 0.5 (empirical)

COD as tetracycline (R4) = COD _tet_ = 20 mg/L x 0.595 x 3 x 0.5 x 1 L = 17.8 mg COD

R1 R2 R3 R4

COD _tet_ 0 g 0 g 0 g 0.02 g

**COD Converted to biomass**

R1 R2 R3 R4

Generated biomass = 0.64 g 0.55 g 0.85 g 0.38 g

Wasted biomass = 1.17 g total (1.8 g/L average and 0.05 L wasted per cycle (13 cycles)) (R1 – R4)

Average microorganism decay rate b_a_ = 0.01 d^-1^ (Rittmann and McCarty, 2012)

Total biomass = generated biomass + wasted biomass + decayed biomass

Total biomass (R1) = 0.64 g + 1.17 g + (0.01 x 1.8 g/L x 1 L (tot. vol.) x 40 d) = 2.53 g

COD conv. to biomass = 1.42 g COD / g MLVSS (Rittmann and McCarty, 2012)

COD converted to biomass (R1) = COD _biomass_ = 2.53 g x 1.42 = 3.59 g COD

R1 R2 R3 R4

Total biomass 2.53 g 2.44 g 2.74 g 2.27 g

COD _biomass_ 3.59 g 3.47 g 3.89 g 3.22 g

**COD Remaining in Solution**

R1 R2 R3 R4

COD _remaining_ 0.20 g 0.30 g 0.23 g 0.30 g

**Total COD Mass Balance**

COD _influent_ = 9.66 g COD (R1 – R4)

Total COD (in) = COD _influent_ + COD _tet_

Total COD (out) = COD _propionate_ + COD _biomass_ + COD _CH4_ + COD _remaining_

R1 R2 R3 R4

Total COD (in) 9.66 g 9.66 g 9.66 g 9.68 g

Total COD (out) 9.49 g 9.53 g 9.56 g 9.69 g

**Table S2.** Sequences of the oligonucleotide primers for qPCR used in this study.

| Primer | | Target Gene | Sequence | Standard curve equation | Annealing Temp. (°C) | Amplicon Size (bp) | Reference |
| --- | --- | --- | --- | --- | --- | --- | --- |
|  | 27-F | 16S rRNA | AGAGTTTGATCCTGGCTCAG | y = -4.00x + 43.8  R^2^ = 0.99 | 57 | 348 | ([Frank et al. 2007](#_ENREF_1)) |
|  | 338-R |  | GCTGCCTCCCGTAGGAGT |  |  |  | ([Amann et al. 1990](#_ENREF_2)) |
| **Ribosomal protection protein genes** | | | |  |  |  |  |
|  | tetW-F | tetW | GAGAGCCTGCTATATGCCAGC | y = -3.49x + 38.9  R^2^ = 0.98 | 60 | 168 | ([Aminov et al. 2001](#_ENREF_3)) |
|  | tetW-R |  | GGGCGTATCCACAATGTTAAC |  |  |  |  |
|  | tetQ-F | tetQ | AGAATCTGCTGTTTGCCAGTG | y = -3.26x + 36.3  R^2^ = 0.99 | 55 | 169 |  |
|  | tetQ-R |  | CGGAGTGTCAATGATATTGCA |  |  |  |  |
| **Efflux pump genes** | | | |  |  |  |  |
|  | tetG-F | tetG | GCAGAGCAGGTCGCTGG | y = -3.42x + 40.0  R^2^ = 0.997 | 60 | 134 | ([Aminov et al. 2002](#_ENREF_4)) |
|  | tetG-R |  | CCYGCAAGAGAAGCCAGAAG |  |  |  |  |
|  | tetZ-F | tetZ | CCT TCT CGA CCA GGT CGG | y = -3.83x + 40.09  R^2^ = 0.99 | 60 | 210 |  |
|  | tetZ-R |  | ACC CAC AGC GTG TCC GTC |  |  |  |  |

**References**

Frank, D.N., St Amand, A.L., Feldman, R.A., Boedeker, E.C., Harpaz, N. and Pace, N.R. (2007) Molecular-phylogenetic characterization of microbial community imbalances in human inflammatory bowel diseases. Proc Natl Acad Sci U S A 104(34), 13780-13785.

Amann, R.I., Binder, B.J., Olson, R.J., Chisholm, S.W., Devereux, R. and Stahl, D.A. (1990) Combination of 16s Ribosomal-Rna-Targeted Oligonucleotide Probes with Flow-Cytometry for Analyzing Mixed Microbial-Populations. Appl Environ Microbiol 56(6), 1919-1925.

Aminov, R.I., Garrigues-Jeanjean, N. and Mackie, R.I. (2001) Molecular ecology of tetracycline resistance: development and validation of primers for detection of tetracycline resistance genes encoding ribosomal protection proteins. Appl Environ Microbiol 67(1), 22-32.

Aminov, R.I., Chee-Sanford, J.C., Garrigues, N., Teferedegne, B., Krapac, I.J., White, B.A. and Mackie, R.I. (2002) Development, validation, and application of PCR primers for detection of tetracycline efflux genes of gram-negative bacteria. Appl Environ Microbiol 68(4), 1786-1793.

**Table S3.** Relative abundance (%) of genera identified as methanogens normalized against the total archaeal community in the anaerobic digesters (R1, R2, R3 and R4: 0, 1 µg/L, 150 µg/L and 20 mg/L of TC-HCl, respectively; R4_B21_ and R4_A21_ represents the samples collected from R4 before and after day 21 respectively). An asterisk (*) denotes that average relative abundance was significantly different (p < 0.05) than that of the control R1. For sample clusters R4_B21_ and R4_A21_, an asterisk (*) indicates a significant difference in relative abundance for that particular genera when compared to each other.

|  | R1 | R2 | R3 | R4 | R4_B21_ | R4_A21_ |
| --- | --- | --- | --- | --- | --- | --- |
| ***Methanobrevibacter*** | 0.7 | 0.5 | 0.9 | 0.9 | 0.9 | 1.0 |
| ***Methanobacterium*** | 14.5 | 14.0 | 13.6 | 13.0 | 14.3 | 11.2 |
| ***Methanosarcina*** | 1.1 | 0.6 | 1.3 | 0.9 | 0.5* | 1.3* |
| ***Methanothrix*** | 61.8 | 63.9 | 62.4 | 61.8 | 65.1 | 57.3 |
| ***Methanoculleus*** | 16.6 | 16.3 | 15.0 | 14.0 | 10.9* | 18.0* |
| ***Methanomassiliicoccus*** | 3.4 | 3.1 | 4.3 | 7.0* | 5.1* | 9.4* |

**B)**

**A)**

**Figure S1.** The dynamic of tetracycline hydrochloric acid (TC-HCl) **A**) in the liquid phase and **B**) in the solid phase in R1-R4

(0 µg/L, 1 µg/L, 150 µg/L, and 20 mg/L of tetracycline hydrochloric acid, respectively). The vertical bars associated with each data point reflect the standard deviation.

**Figure S2.** Comparison of the COD profiles over time in R1-R4

(0 µg/L, 1 µg/L, 150 µg/L, and 20 mg/L of tetracycline hydrochloric acid, respectively).

The vertical bars associated with each data point reflect the standard deviation.

**Determination of tetracycline concentrations**

*Solid phase extraction of TC-HCl*

After centrifugation, the supernatant was filtered through a 0.22 µm cellulose acetate syringe filter and stored in an amber glass bottle, and the pellets were stored in a freezer at -80 ºC. 20 mL of Na_2_EDTA-McIlvaine buffer, prepared by mixing 19.2 g of citric acid, 17.75 g of Na_2_HPO_4_ and 60.5 g of Na_2_EDTA in 1.625 L of deionized water, was added to the amber glass bottle prior to solid-phase extraction (SPE).

To determine the TC-HCl in the solid phase, the collected pellets were conducted as follows. Briefly, the centrifuged biomass was lyophilized with a freeze-dryer, and extracted with 20 mL of 1:1 (v/v) of EDTA-SPE (prepared by mixing 12.14 g of NaH_2_PO_4_∙H_2_O, 37.2 g of Na_2_EDTA∙H_2_O, and 0.82 mL of H_3_PO_4_ in 1 L deionized water) and acetonitrile. The suspension was vortexed for 5 s, ultrasonicated in an ice-water bath for 15 min, and then centrifuged at 3600 *g* and 4 °C for 20 min. The supernatant was collected in a clean 50 mL centrifuge tube. With 10 mL of the 1:1 (v/v) of EDTA-SPE and acetonitrile, the same lyophilized samples were subjected to an additional two rounds of extraction, and the supernatant obtained from all three rounds of extraction was combined. The combined supernatant was then directly filtered through a 0.45 µm syringe nylon membrane filter, and diluted with deionized water prior to adding Na_2_EDTA-McIlvaine buffer in a 3:2 v/v ratio, enabling < 5% of acetonitrile in the total extracted matrix.

All samples prepared as described above were individually added with 20 µg/L of tetracycline-d6 (VWR, UK) surrogate. SPE was then performed on a Dionex Autotrace 180 SPE equipment (Thermo Fisher Scientific, Waltham, MA). Briefly, the SPE cartridge (Oasis HLB, 500 mg) was conditioned with methanol, 10 mM trifluoroacetic acid (TFA) and deionized water, consecutively, at a flowrate of 15 mL/min. The sample was then loaded with a flowrate of 1 to 3 mL/min d(Aydin et al. 2015)epending on the sample volume. Once the sample loading was completed, the cartridge was consecutively rinsed with 10 mL methanol/deionized water (5:95 v/v) and 10 mL deionized water. The cartridge was then dried under a gas stream for 60 min, and eluted twice with 5 mL of 0.1% formic acid in methanol at 1 mL/min. The eluted solvent was collected, and evaporated under a nitrogen gas stream to concentrate to a final volume of 0.5 ml, prior to LC-MS/MS analysis for TC-HCl concentration.

*Quantitation of TC-HCl*

The liquid chromatography (LC) was performed on HPLC 1260 Infinity (Agilent Technologies, Santa Clara, CA) fitted with a Luna®-C18(2) column and SecurityGuard^TM^ cartridges (Phenomenex, Torrance, CA). The gradient mobile phase was composed of water (phase A) and methanol (phase B). Both phases were acidified with 0.1% (v/v) of formic acid. The isolated sample was further detected by Tandem Mass Spectrometry QTRAP 5500 (Sciex, Framingham, MA). A series of standard solution (2 to 50 µg/L) for TC-HCl and isotope-TC were prepared. Similar to the sample, all the standard solution for TC and isotope TC were conducted with the SPE before being detected by LC-MS/MS. A calibration curve was then generated by plotting the peak areas against their corresponding amounts of standard solution.

**Microbial community analysis**

*PCR amplification for Illumina MiSeq sequencing*

The DNA from pellets was extracted according to the PowerSoil DNA Isolation kit (Mo Bio, Laboratories, Inc, Carlsbad, USA). 16S rRNA gene was amplified with the primer pair 515F (5′- Illumina overhang- GTGYCAGCMGCCGCGGTAA- 3′) and 907R (5′- Illumina overhang CCCCGYCAATTCMTTTRAGT- 3′). After the PCR amplicons being clean-up with AMpure XP beads (Beckman Coulter, CA, USA), an Index PCR was further performed to barcode the microbial sample via the Nextera XT Index Kit (Illumina Ina, San Diego, CA, USA). With a clean-up of the Indexed PCR amplicons via AMpure XP beads, equimolar amount of the purified DNA sample was pooled together and submitted to KAUST core lab for Illumina MiSeq sequencing.

*RDP Classifier- and OTU-based analysis methods*

The sequences without the chimera were analyzed by two methods. Briefly, the first approach involved assigning sequences to a taxonomic hierarchy to the genus level at a 95% confidence interval using the Ribosomal Database Project (RDP) Classifier and adjusting the calculated relative abundances based on 16S rRNA gene copies per cell of the genera identified. The second approach utilized the sorting of sequence files to identify and cluster all identical sequences to create unique operational taxonomic units (OTUs) for each of the sequence clusters at a 97% 16S rRNA gene similarity confidence level. These OTUs were then normalized to the total number of chimera-removed sequences in each sample. OTUs with an average relative abundance of greater than 0.3% were blasted against the NCBI nucleotide database using the BLASTN algorithm to determine identity similarity data of the closest matching bacterial or archaeal species at an E-value < 10^-5^.
